# TIGA-CUB – manualised psychoanalytic child psychotherapy versus treatment as usual for children aged 5–11 years with treatment-resistant conduct disorders and their primary carers: study protocol for a randomised controlled feasibility trial

**DOI:** 10.1186/s13063-017-2166-2

**Published:** 2017-09-15

**Authors:** Elizabeth Edginton, Rebecca Walwyn, Kayleigh Burton, Robert Cicero, Liz Graham, Sadie Reed, Sandy Tubeuf, Maureen Twiddy, Alex Wright-Hughes, Lynda Ellis, Dot Evans, Tom Hughes, Nick Midgley, Paul Wallis, David Cottrell

**Affiliations:** 1 Child Oriented Mental health Interventions Centre (COMIC), Leeds and York Partnership NHS Foundation Trust, University of York, IT Building, Innovation Way, York, YO10 5NP UK; 2 Leeds Clinical Trials Research Unit, University of Leeds, Leeds, UK; 3 Leeds Institute of Health Sciences, University of Leeds, Leeds, UK; 4 Northern School of Child and Adolescent Psychotherapy (NSCAP), Leeds and York Partnership NHS Foundation Trust, Leeds, UK; 5 City of York Council, York, UK; 6 General Adult Psychiatry, Leeds and York Partnership NHS Foundation Trust, Leeds, UK; 7 Child Attachment and Psychological Therapies Research Unit (ChAPTRe), Anna Freud National Centre for Children and Families, London, UK; 8 The Winnicott Centre, CAMHS Central Manchester University Hospitals NHS Foundation Trust, Manchester, UK

**Keywords:** Randomised controlled trial, Psychoanalytic child psychotherapy, Conduct disorders, Treatment-resistant, Inter-generational attachment

## Abstract

**Background:**

The National Institute for Health and Care Excellence (NICE) recommends evidence-based parenting programmes as a first-line intervention for conduct disorders (CD) in children aged 5–11 years. As these are not effective in 25–33% of cases, NICE has requested research into second-line interventions. Child and Adolescent Psychotherapists (CAPTs) address highly complex problems where first-line treatments have failed and there have been small-scale studies of Psychoanalytic Child Psychotherapy (PCP) for CD. A feasibility trial is needed to determine whether a confirmatory trial of manualised PCP (mPCP) versus Treatment as Usual (TaU) for CD is practicable or needs refinement. The aim of this paper is to publish the abridged protocol of this feasibility trial.

**Methods and design:**

TIGA-CUB (Trial on improving Inter-Generational Attachment for Children Undergoing Behaviour problems) is a two-arm, pragmatic, parallel-group, multicentre, individually randomised (1:1) controlled feasibility trial (target *n* = 60) with blinded outcome assessment (at 4 and 8 months), which aims to develop an optimum practicable protocol for a confirmatory, pragmatic, randomised controlled trial (RCT) (primary outcome: child’s behaviour; secondary outcomes: parental reflective functioning and mental health, child and parent quality of life), comparing mPCP and TaU as second-line treatments for children aged 5–11 years with treatment-resistant CD and inter-generational attachment difficulties, and for their primary carers. Child-primary carer dyads will be recruited following a referral to, or re-referral within, National Health Service (NHS) Child and Adolescent Mental Health Services (CAMHS) after an unsuccessful first-line parenting intervention. PCP will be delivered by qualified CAPTs working in routine NHS clinical practice, using a trial-specific PCP manual (a brief version of established PCP clinical practice). Outcomes are: (1) feasibility of recruitment methods, (2) uptake and follow-up rates, (3) therapeutic delivery, treatment retention and attendance, intervention adherence rates, (4) follow-up data collection, and (5) statistical, health economics, process evaluation, and qualitative outcomes.

**Discussion:**

TIGA-CUB will provide important information on the feasibility and potential challenges of undertaking a confirmatory RCT to evaluate the effectiveness and cost-effectiveness of mPCP.

**Trial registration:**

Current Controlled Trials, ID: ISRCTN86725795. Registered on 31 May 2016.

**Electronic supplementary material:**

The online version of this article (doi:10.1186/s13063-017-2166-2) contains supplementary material, which is available to authorized users.

## Background

Conduct disorders (CD) are ‘repetitive and persistent patterns of antisocial, aggressive or defiant behaviour that amount to significant and persistent violations of age-appropriate social expectations’ [1]. They affect 5% of 5–10-year-olds (187,500 children in England and Wales, based on 2011 census data), with prevalence expected to rise [2, 3]. Pre-adolescent CD risk particularly adverse outcomes, including poor educational achievement, teenage pregnancy, substance abuse, criminality, long-term physical and mental illness, unemployment, homelessness, domestic violence, and poor subsequent parenting [4–13]. Children’s CD impact substantially on the NHS, constituting 30% of GP child consultations and 45% of child community health referrals [1]. Long-term public service costs can be ten times higher (£70 k) for those with childhood CD than for those without, and related crime costs £22.5 billion annually [14–18]. CD are, therefore, an important, expensive, and growing problem.

There is clear evidence that many parenting programmes deliver effective outcomes [1]. However, our scoping study, funded by the National Institute of Health Research (NIHR) Flexibility and Sustainability Funding (FSF) and conducted in five Child and Adolescent Mental Health Services (CAMHS) (*n* = 145)(Edginton E, Twiddy M. Scoping study in regional CAMHS in the north of England on children aged 5-11 with conduct disorders, unpublished) showed that evidence-based protocols are often not followed. Children and Young People’s Improving Access to Psychological Therapies (CYP-IAPT – a national programme which aims to develop an improved CAMHS) should help with this, but even evidence-based programmes do not benefit 25–33% of families, and 30–40% drop out [1, 19, 20]. The long-term costs of treatment-resistant CD are extremely high, and if parent training fails, there are few alternatives. Cognitive-behavioural and/or social learning programmes are only recommended for children aged 9+ years [1], and multisystemic interventions only for children aged 11+ years [1]. These options either involve a major component of parent training, which will already have failed for our intended patient group, or demonstrate small effect sizes, suggesting that these options will be unsuitable for treatment-resistant CD, or are more resource-intensive and intended for older children [1]. National Institute for Health and Care Excellence (NICE) has, therefore, requested research on urgently needed, second-line treatments [1].

Psychoanalytic Child Psychotherapy (PCP) for treatment-resistant CD is supported by theoretical and pragmatic research as well as by clinical experience. Large meta-analyses on links between attachment and children’s CD have found correlations between insecure (and particularly, disorganised) attachment and subsequent behaviour problems [21–24]. Other studies have found links between children’s CD and primary-carer (particularly maternal) attachment difficulties [25–28], and between children’s disorganised attachment and parental trauma [29]. Both secure and insecure attachment are liable to inter-generational transmission [29], with one meta-analysis finding a match between mother and child secure-insecure attachment styles of 75% (*κ* = .49) [30]. In our scoping study of children with behaviour difficulties in CAMHS, 60% of mothers indicated potential inter-generational attachment difficulties (Edginton E, Twiddy M. Scoping study in regional CAMHS in the north of England on children aged 5-11 with conduct disorders, unpublished). These are linked to problems in reflective functioning, i.e. understanding behaviour in terms of underlying thoughts and feelings [31, 32], and to adult mental health problems [33, 34], which can contribute to children’s CD [1, 35]. Nevertheless, secure attachment can be ‘earned’ via positive experiences post infancy [36–38]. Child and Adolescent Psychotherapists' (CAPTs’) clinical experience that families of children with CD often have inter-generational attachment difficulties is thus endorsed by both theoretical and empirical research.

It is widely accepted that CAPTs address highly complex problems – including inter-generational attachment difficulties – where first-line treatments have failed [39, 40]. CAPTs are trained to work with children and their primary carers, to recognise adult mental health difficulties, and to refer parents/carers to GPs and adult services, where appropriate [41]. There have been several small studies of PCP for CD. A retrospective study of children in intensive and non-intensive psychotherapy (*n* = 763) including children with CD found that 49% of children overall made clinically significant improvements in levels of functioning on the Hampstead Child Adaptation Measure (HCAM) [42], while a retrospective case-control study comparing children with a disruptive disorder (*n* = 135) and those with an emotional disorder found significant improvement in functioning in both the disruptive and emotionally disordered groups [43]. A naturalistic study (*n* = 9) found similar improvements in overall functioning and externalising behaviour using the Global Assessment of Functioning scale (GAF) and the Child Behavior Checklist (CBCL) and CBCL Teacher Report Form (CBCL-TRF) [44]. A controlled trial (*n* = 26) resulted in clinically significant improvements on the Impairment Score for Children and Adolescents (IS-CA) in 31% of children, compared with 8% in the waiting list control, but no significant difference on the CBCL [45]. A randomised controlled trial (RCT) (*n* = 69) found Family Therapy and Child Psychotherapy equally superior to the recreational control treatment group using the CBCL [46]. However, these studies all had small samples and were not conducted in UK CAMHS.

The PCP intervention used in the TIGA-CUB (Trial on improving Inter-Generational Attachment for Children Undergoing Behaviour problems) feasibility study is a systematic, manualised, brief version of existing clinical practice. It comprises 12 sessions for the child and 12 sessions for the primary carer (and any other of the child’s parents/carers who wish to attend). The term ‘primary carer’ denotes the adult – i.e. parent or kinship carer – the parents/carers consider to be the most involved in parenting the child. The ‘change hypothesis’ is that a regular, predictable, and intensive positive attachment experience with the CAPT for the child is likely directly to improve the child’s behaviour, and that direct work with the primary carer and any other parent/kinship carer who wishes to attend is likely to enhance parental reflective functioning [47, 48], improving understanding of the emotional distress that the child is communicating via the problematic behaviour [44, 49]. The work is also likely to empower primary carers, making them feel less stressed, and to help them gain greater control over parenting behaviour through understanding their own experiences of having been parented and of how their child’s behaviour might trigger their own complex and possibly traumatic childhood memories [50]. The intervention might also begin to assist primary carers to acknowledge mental health difficulties and to access adult services, where appropriate [40]. It is anticipated that primary carer benefits will result in a more sympathetic approach to the child to which the child will respond, thereby promoting a positive cycle of change.

This paper describes the protocol for the feasibility trial evaluating manualised Psychoanalytic Child Psychotherapy (mPCP) compared with Treatment as Usual (TaU) within CAMHS. In the absence of a clear evidence base at the time of our scoping study and when funding was obtained, TaU tended to be highly heterogeneous, including interventions which ranged from generic non-specific parent or child work and parenting programmes through to Play Therapy, Family Therapy, and non-manualised, long-term PCP. Our comparator is, therefore, heterogeneous TaU rather than a specific, homogeneous alternative, both because the latter is not currently practicable in CAMHS, and because one or even two comparators would answer the explanatory, but not the pragmatic, question of interest to the NHS.

### Aims and objectives

The aims of this trial are:

1. To assess the feasibility of conducting, and
2. To inform the design of, a large-scale, confirmatory RCT of second-line mPCP versus TaU for children aged 5–11 years with treatment-resistant CD and their primary carers, so as to improve the range of treatment options and long-term outcomes for this hard-to-treat patient group.

The objectives of the trial are:

1. To establish procedures for screening children referred to, or re-referred within, CAMHS with CD and their primary carers, and to estimate the recruitment rate and the proportion of primary carers with mental health difficulties
2. To assess acceptability to clinicians and primary carers of randomising to mPCP or TaU.
3. To collect baseline and 4-month blinded follow-up data (and 8-month follow-up data for those recruited in the first 4 months of the trial), to estimate likely follow-up rates and data quality for the confirmatory trial
4. To collect data on treatments received within CAMHS across trial arms to characterise co-interventions and TaU over time
5. To assess the feasibility of, and best methods for, collecting data from various sources (primary carers, children, and teachers), including national routine data sets as they become available during the lifetime of the trial
6. To assess the feasibility of, and best methods for, collecting adverse events relating to the child or primary carer
7. To identify the most appropriate way to collect quality of life and cost data to assess short and long-term cost-effectiveness
8. To assess the feasibility of keeping researchers blinded, by monitoring the rate and reasons for unblinding
9. To establish procedures for assessing treatment attendance and adherence to the intervention
10. To confirm the variability and clustering of outcomes by clinician to inform sample size calculations for the confirmatory trial.

The full protocol (version 3.2, 20 April 2017) is available on request from the corresponding author.

## Methods and design

### Design

**Fig. 1:**
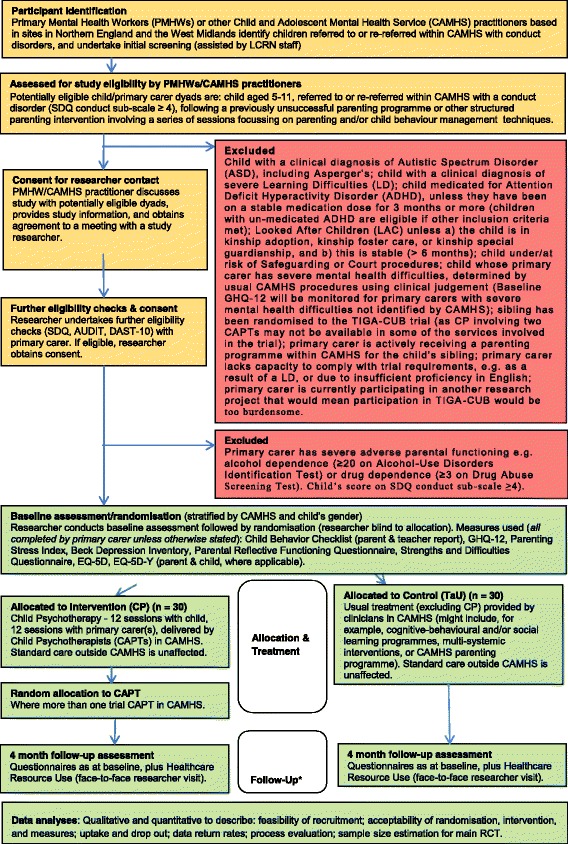
TIGA-CUB trial flow diagram

TIGA-CUB is a multicentre, two-arm, pragmatic, parallel-group, individually randomised controlled feasibility trial. A summary of the trial design is included as a flow diagram below (see Fig. 1).

### Setting

CAMHS in (1) Bradford and Keighley, (2) Dudley and Walsall, (3) Sheffield, and (4) Wakefield and Castleford were selected to participate to maximise generalisability and to provide a good test of the feasibility of recruitment. Service configurations vary considerably across these settings, with all CAMHS using a triage system on first referral, but with different subsequent internal pathways, and with some CAMHS having only one CAPT while others have up to five.

### Eligibility criteria

Participant eligibility will be assessed in stages. Inclusion and exclusion criteria will first be assessed by CAMHS practitioners involved in screening referrals. The final two exclusion criteria (primary-carer alcohol and drug dependence) will be assessed by the researcher when contacting participants who have consented to researcher contact, as will confirmation of a clinical level of CD.

Inclusion criteria:

- Child aged 5–11 years old at baseline
- Presenting to CAMHS, or re-referred within CAMHS, with a clinical level of CD ≥ 4 on the Strengths and Difficulties Questionnaire (SDQ) [51] conduct subscale
- Child’s current primary carer has been offered a first-line group or individual parenting programme or other structured parenting intervention in primary care or within CAMHS, involving a series of structured sessions focussing on parenting and/or child behaviour management techniques, and has attended at least one session, but the child’s CD persists and the child has been referred to CAMHS or re-referred within CAMHS for further treatment.

Exclusion criteria:

- Child with a clinical diagnosis of autistic spectrum disorder (ASD), including Asperger’s syndrome
- Child with a clinical diagnosis of severe learning difficulties (LD)
- Child medicated for attention deficit hyperactivity disorder (ADHD), unless they have been on a stable medication dose for 3 months or more (children with un-medicated ADHD are eligible if other inclusion criteria are met)
- Looked After Child (LAC), unless they are in a stable (at least 6 months) kinship adoption, kinship foster care or kinship special guardianship relationship with their carer
- Child under/at risk of safeguarding or court procedures
- Child whose primary carer has severe mental health difficulties, determined by usual CAMHS procedures using clinical judgement (baseline General Health Questionnaire (GHQ-12) [52] will be monitored for primary carers with severe mental health difficulties not identified by CAMHS)
- Sibling has been randomised to TIGA-CUB (avoidance of potential contamination)
- Primary carer is actively receiving a parenting programme within CAMHS for the child’s sibling
- Primary carer lacks capacity to comply with trial requirements, e.g. as a result of a LD, or due to insufficient proficiency in English
- Primary carer is currently participating in another research project that would mean participation in TIGA-CUB would be too burdensome
- Primary carer has severe adverse parental functioning, e.g. alcohol dependence (≥20 on Alcohol-Use Disorders Identification Test) (AUDIT-C) [53] or drug dependence (≥ 3 on the Drug Abuse Screening Test) (DAST-10) [54], determined by the trial researcher on initial screening visit.

### Participant identification and recruitment

Due to CAPT clinical practice and availability, it is anticipated that recruitment will take place in line with school terms and half terms where possible, resulting in recruitment of up to five cohorts of participant dyads during the recruitment period. Recruitment will be initiated after referral to, or re-referral within, CAMHS. All referrals aged between 5 and 11 years with a potential CD will be screened. Once a potential primary carer-child dyad has been identified and CAPT capacity has been confirmed, the local CAMHS practitioner will introduce the trial to them. If the primary carer is interested in participating, the primary carer and child will be provided with TIGA-CUB Information Leaflets and consent will be gained for initial researcher contact. In addition, as part of the process evaluation for the trial, primary carers will receive a questionnaire gauging their understanding of the trial and asking about their views on the recruitment process, and will be asked if they would be willing to consent to a process evaluation researcher contacting them subsequently to collect more detailed feedback. Responses will be reviewed so that recruitment processes can be refined for a confirmatory trial.

Where consent is given for initial researcher contact, the primary carer will be contacted and a meeting scheduled either at the primary carer’s home or at another appropriate location to explain the trial in greater detail, establish full trial eligibility, obtain consent, and undertake the baseline assessment. The dyad will have been given at least 24 h to read and digest the Information Leaflets previously provided, and will have had the opportunity to discuss them with their family and/or other health care professionals (e.g. GP) if they so wish, before being asked whether they would be willing to take part in the trial.

All primary carers will be asked to complete the AUDIT-C and DAST-10, plus the SDQ. Formal consent will not be required prior to completion of these measures, as completion will constitute implied consent. Those who decline at this stage will be offered TaU for their child. Those who complete these measures but score outside of the thresholds specified in the inclusion criteria will not be eligible to take part, and will also be offered TaU.

Where dyads fulfil all the eligibility criteria, the primary carer will be invited to provide informed written consent. Given the age of the children involved in the trial (5–11 years old), their formal consent will not be obtained, and the primary carer will provide consent for their inclusion. However, as per the Health Research Authority (HRA) guidance for research involving children, children will still be involved in the decision-making process with the right to voice their views and to influence the resulting decision, and will be provided with information that matches their capacity. Should the child actively object to taking part in the trial, then they will not be included, and reasons for non-participation will be recorded, where provided.

### Randomisation

Following confirmation of eligibility and consent, and subsequent to the baseline assessment being performed, dyads will be randomised by the trial researcher via the Clinical Trials Research Unit’s) (CTRU’s) automated 24-h randomisation system. Dyads will be randomised (1:1 basis) to receive mPCP or TaU. A computer-generated minimisation algorithm incorporating a random element will be used, stratifying for (1) site and (2) gender of child. If a dyad is randomised to mPCP in a CAMHS clinic in which multiple CAPTs are available to deliver the intervention, dyads will also be randomly allocated to the CAPT who will deliver the child’s intervention, with an allocation ratio proportional to CAPT availability, to avoid selection bias. Where only one CAPT works in a CAMHS, the CAPT will automatically be selected as the delivering CAPT. The CTRU will inform the CAMHS, but not the researcher (to preserve blinding), of the outcome of randomisation. The CAMHS will contact the primary carer to explain the outcome of the randomisation and to make an appointment for initiation of treatment. Therefore, participants and CAPTs will, of necessity, be aware of treatment allocation.

### Interventions

#### Manualised Psychoanalytic Child Psychotherapy (mPCP)

Qualified CAPTs who are current members of the Association of Child Psychotherapists (ACP), working in CAMHS, and trained in the trial intervention will deliver mPCP (referring to both sessions for the child and work with the primary carer and any other of the child’s parents/carers wishing to attend), using the TIGA-CUB manual. The manual is sufficiently flexible to accommodate the range of situations likely to be encountered in the trial, and will be reviewed (and updated if necessary) at the end of the feasibility trial by the Trial Management Group (TMG) to ensure that it is appropriate for a confirmatory trial.

Children will attend mPCP sessions of approximately 50-min duration each, delivered over 12 weeks at approximately weekly intervals (excluding breaks of no more than 2 weeks at normal school holidays). This will equate to a total of up to 12 sessions. The primary carer will attend parent work sessions of similar number, duration, and spacing. It is planned that the child and primary carer, respectively, will be seen separately either by the same CAPT or by two different CAPTs. The reason for this decision will be recorded. Where the child and primary carer are seen by the same CAPT, the sessions will, of necessity, take place at different times, but will take place in the same week as far as is practically possible. If the child and primary carer are seen by two different CAPTs, the sessions will either take place at the same time, or will take place at different times within the same week, where possible. Where the child and primary carer are seen by two different CAPTs at different times, the primary carer sessions will commence prior to those of the child. Following PCP practice, the day, time, and location of all sessions will be kept consistent unless there are exceptional circumstances, and the intervention will usually commence no less than 3 weeks prior to an end-of-term or half-term break so as to facilitate the establishment of the therapeutic alliance. A holiday break will nevertheless be incorporated where possible, to give the child and primary carer a ‘practice run’ at a therapeutic ending.

As with TaU, it is anticipated that some participants will miss sessions because of dropout (intermittent or complete) or mutually agreed early termination of treatment. Conversely, some may go on to receive more sessions, where this is deemed clinically appropriate, although it is anticipated that this will be the exception rather than the rule. In line with usual CAMHS practice, each local CAMHS will identify a named case manager for the CAPT(s) to liaise with, to provide assurance with regard to clinical governance.

All eligible CAPTs working within a recruiting CAMHS will be trained and supervised to deliver mPCP in the trial. CAPTs will receive appropriate training in the use of the manual and adherence to it, delivered by the chief investigator, and other relevant members of the TMG if necessary. Small-group supervision of participating CAPTs will be conducted approximately fortnightly, in person except in exceptional circumstances, by supervisors experienced in working with children with CD and in the use of the manual who will themselves attend termly supervision, to facilitate adherence to the manual and quality of care. Supervising CAPTs will be trained by appropriate members of the TMG. These arrangements will ensure a fair test of mPCP by using qualified CAPTs receiving regular clinical supervision, which is accepted best practice in CAMHS and in the ACP.

#### Treatment as Usual (TaU)

TaU will comprise the usual care offered by local CAMHS to children aged 5–11 years with CD. This treatment is likely to be highly diverse and may involve group and/or individual child and/or primary carer and/or family based work, delivered by a range of practitioners from a variety of professional backgrounds and theoretical orientations. The duration of TaU in CAMHS will vary considerably, but our scoping study demonstrated that the average amount of time spent in CAMHS is likely to be at least 33 weeks (Edginton, E, Twiddy, M. Scoping study in regional CAMHS in the north of England on children aged 5-11 with conduct disorders, unpublished). The confirmatory trial will be pragmatic, involving several collaborating CAMHS, and so currently, it would not be possible to specify what TaU should be. We will, therefore, monitor what it is in this feasibility trial and how this varies across CAMHS. Our scoping study will be used to inform data collected, and the duration of time that families spend within CAMHS will be recorded. It is, nevertheless, expected that CAMHS clinicians will be working in line with best practice, as per the NICE guidelines for children with CD [1]. In addition, in line with current best practice, it is expected that practitioners delivering TaU will be in receipt of supervision, and this will also be monitored as part of the trial. CAPTs at sites will not provide mPCP, non-manualised PCP, or TaU to dyads allocated to TaU and will, therefore, have no contact with TaU participants.

#### Both groups

All child and primary carer participants will be seen within CAMHS. Clinicians in both arms of the trial will have access to local Child Psychiatrists if medication or hospitalisation needs to be considered for any of the children participating in the trial. Routine care provided outside CAMHS will be unaffected for both arms.

### Data collection

#### Participant data

Participant assessments will be undertaken at the following time points:

- Baseline (prior to randomisation)
- 4 months post randomisation
- 8 months post randomisation (for participants recruited in the first 4 months of the trial).

A window of up to 2 weeks prior to randomisation will be recommended for baseline data collection (ideally, baseline and randomisation will occur on the same day) and plus or minus 2 weeks from the target follow-up date for outcome data. The feasibility of these windows will be recorded (see Table 1).

**Table 1:** Summary of assessments

Assessment (and involvement*)	Timeline (months post randomisation)			
	Screening	Baseline	4 months	8 months
Eligibility and consent				
• Eligibility (inclusion criteria assessed by clinician)	x	x		
• Consent (P)		x		
Background, demographics, interview data (C, P, R)				
• Personal details (e.g. contact details)		x		
• Current comorbid physical/mental health		x	x	x
• Psychotropic medications		x	x	x
• Family composition		x	x	x
• Difficulties since randomisation			x	x
Clinical data (P, R, CSO)				
• Therapy details (CSO)			x	
• Therapist supervision details (therapist/supervisor)			x	
• Psychotropic medications details (CSO)			x	
• Referrals to other services (CSO)			x	
• Re-referrals to, or referrals within, CAMHS (CSO)			x	
• Baseline therapist data		x		
• Serious adverse event reporting	Ongoing collection			
Questionnaires				
• AUDIT-C (P)	x			
• DAST-10 (P)	x			
• SDQ (P)	x		x	
• EQ-5D-Y (P, proxy-completion and C, where able)		x	x	x (just P)
• EQ-5D (P)		x	x	
• CBCL (P)		x	x	x
• CBCL-TRF (T)		x	x	
• GHQ-12 (P)		x	x	
• Parenting Stress Index (P)		x	x	
• Beck Depression Inventory (P)		x	x	
• Parental Reflective Functioning Questionnaire (P)		x	x	x
• Healthcare Resource Use (P)			x	
Process evaluation				
• Primary Carer Recruitment Feedback Questionnaire (P)	x			
• ‘End of trial’ survey (P)			x	
• Semi-structured interview (sample of P)			x	
• Child Psychotherapist Focus Groups (CAPT)			**	

*C child, *P* primary carer, *R* researcher, *CSO* clinical studies officer (from case notes), *T* teacher, *CAPT* child psychotherapist; **the focus group with CAPTs will be conducted after the final sessions of CP have been delivered

*AUDIT-C* Alcohol-Use Disorders Identification Test, *CBCL* Child Behavior Checklist, *CBCL-TRF* Child Behavior Checklist-Teacher Report Form, *DAST-10* Drug Abuse Screening Test, *EQ-5D* EuroQol 5 Dimensions, *EQ-5D-Y* EuroQol 5 Dimensions Youth, *GHQ-12* General Health Questionnaire, *SDQ* Strengths and Difficulties Questionnaire

At baseline and 4 months post randomisation, the following questionnaires will be used:

- Child Behavior Checklist (CBCL and CBCL-TR) [55, 56] (parental rating of child’s problem behaviours and competencies, and teacher rating of academic achievement and behaviour in class, in addition to attendance and performance)
- General Health Questionnaire 12 (GHQ-12) [52] (parental mental health)
- Parenting Stress Index (PSI) [57] (primary carer rating of their own functioning, functioning of child, and level of stress in the primary carer-child relationship)
- Beck Depression Inventory (BDI) (v2.0) [58] (screening instrument for adult depressive symptoms)
- Parental Reflective Functioning Questionnaire (PRFQ) [59–61] (measure of reflective functioning)
- EuroQol 5 Dimensions (EQ-5D™) (3-level) [62] (parental health-related quality of life)
- EuroQol 5 Dimensions Youth (EQ-5D-Y™) (3-level) (to measure child’s health-related quality of life as reported by the child via face-to-face researcher administration when the child is able to report for themselves, or by the primary carer as a proxy-respondent where the child is unable or unwilling to do so)

Questionnaires will be completed during researcher visits to participants’ homes, or failing that, by telephone or by post if necessary. Additionally, at baseline and at 4-month follow-up, the researcher will collect data from the primary carer on:

- Current comorbid physical/mental health
- Current psychotropic medications (methylphenidate, other specified)
- Current family composition (i.e. who the child lives with)

At 8 months post randomisation, participants providing data will be asked to complete specific measures (CBCL, PRFQ, EQ-5D-Y) over the telephone in the first instance, or via a face-to-face visit or by post if necessary, to maximise follow-up data completion. The child’s school teacher will be asked to complete the CBCL-TR by post at baseline and 4 months post randomisation only.

##### Treatment and other data

Baseline care provider data will be collected from clinicians delivering treatment in both arms of TIGA-CUB to provide details of the treating clinician’s job title, band, length of time working in CAMHS, professional qualifications and additional training in specific therapeutic techniques. The following data covering the period from randomisation up to 4 months post randomisation will be collected from the CAPTs/CAMHS team (or by the researcher or authorised individuals from the research team with appropriate access), and from the CAPT clinical supervisors:

- Care pathway details (type, sessions offered and attended, including sessions where participants did not attend (DNAs) or could not attend (CNAs), dates and duration of sessions, CAMHS practitioners involved, family members involved, and mutually agreed end date)
- Re-referrals to, or re-referrals within, CAMHS
- Referral to other services (including mental health, social, criminal, education, etc.)
- A checklist of expected adverse events (based on those listed in this protocol), with space to specify others not listed
- Supervision details (intervention arm only).

All participants who enter TIGA-CUB will be considered part of the intention-to-treat (ITT) population and efforts will be made to follow them up whenever appropriate.

### Process evaluation

The process evaluation aims to understand the functioning of the intervention and to inform the development of a confirmatory trial protocol by identifying:

- Acceptability of recruitment processes, and reasons for non-consent to trial participation
- Reasons for treatment dropout or withdrawal from trial processes (i.e. from further data collection, researcher follow-up visits or telephone or postal questionnaire follow-up)
- Acceptability of data collection measures and timing of data collection (i.e. burden)
- Possible adverse reactions to taking part in the trial
- CAPT, primary carer (and, if possible, child) perspectives on aspects of the intervention considered to be effective, and perceptions of mechanisms of change
- Processes and challenges of implementing the intervention from the perspective of the CAPTs involved.

A questionnaire will be given to all eligible participants during recruitment. Quantitative data will be collected on primary carers’ characteristics, and on their views of recruitment. At the end of the individual participant’s involvement in the trial, a second brief questionnaire will be sent to gather their views on taking part, on the intervention or other treatment received, and on the trial (except those participants withdrawing from postal questionnaires). If applicable, data will be collected on reasons for treatment dropout and/or for withdrawing from the trial. Free-text responses will be used to allow participants to provide additional feedback about their experiences of taking part in the trial, in addition to the closed questions used in the survey.

Qualitative data on participants’ experiences of the trial will be collected via semi-structured interviews. Consent for interview will be obtained separately from overall trial consent. Feedback will be sought from the following groups:

- Decided against participation when trial presented to them
- Agreed to participate in the trial and randomised
- Declined allocated treatment; dropped out of treatment and/or withdrew from the trial (but not the process evaluation).

In-depth, semi-structured qualitative interviews will be conducted (to data saturation – expected to be about 15 primary carers). Purposive sampling will be used to ensure a wide range of primary carer views, including where possible treatment completers, treatment dropouts, and those declining trial participation. For maximum variation, we will sample on child’s age and gender, and degree of CBCL change, where possible. We will also seek to recruit across centres to identify any centre-level differences. Interviews will seek to maximise understanding of the experiences of those taking part in the trial and, where applicable, their views of the intervention. Depending on age and willingness to participate, attempts will be made to gain some feedback from children (what they liked or did not like), with their primary carer present.

A topic guide will be used to guide the interview discussion and will ask about why participants took part (or not), the recruitment and randomisation process, their experiences of participation, concerns about participation, and burden of involvement. Interviews are expected to last 45 to 60 min. Interviews will be undertaken by a researcher with specific training and/or experience in conducting in-depth, exploratory interviews.

The timing of contact will vary between participant groups:

- Those recruited within the first 3 months of the recruitment period will be contacted after the end of treatment and consent will be sought for interview approximately 8 months after randomisation, to capture longer-term views of the treatment and to assess the burden of data completion on participants
- Those recruited within the last 4 months of the trial will be contacted after, and interviewed within, 2 months of end of therapy to capture the experiences of treatment and recruitment
- Those who decline trial involvement will be contacted within a month of declining the trial.

All CAPTs taking part in the trial will be invited to take part in a focus group discussion to talk about their experiences of using mPCP. The focus group will take place at the end of the trial once all dyads have completed mPCP. A topic guide will be used to explore views about trial design, factors enabling or inhibiting successful recruitment, intervention delivery, and data collection. Participants will also be asked how mPCP fits with their usual clinical practice, aspects of mPCP considered effective/perceived mechanisms of change, and unanticipated or adverse impacts on services or dyads.

### Health economics

This component is included to check the completeness and ability to obtain resource-use data from dyads about both NHS and private costs and quality of life. Wherever possible, unit costs for resources will be obtained from national sources such as the *British National Formulary* (BNF) [63] and the Personal Social Services Research Unit (PSSRU) Costs of Health and Social Care [64]. NHS and Social Service resource use will be identified through direct observation of the treatment provided within the feasibility trial and through the structured questionnaire for collection of data on all other service use. We will assess the level of completeness of EQ-5D-Y as reported by the child and by the primary carer and evaluate any discrepancies using Quality-adjusted Life Years (QALYs) as the outcome.

### Outcomes

Outcomes relate to feasibility of recruitment, therapeutic delivery, retention in treatment, and outcome data collection:

Recruitment methods, uptake, and follow-up: (1) number of dyads screened for eligibility, (2) proportion of carers from eligible dyads consenting to the trial, (3) proportion of consenting dyads randomised, (4) reasons for non-participation, and (5) proportion of randomised dyads completing the trial, number of withdrawals from follow-up data collection, reasons for withdrawal, number of losses to follow-up.

Therapeutic delivery: (1) proportion of dyads randomised to the intervention arm, proportion randomised to, and seen by, the allocated CAPT; proportion of dyads successfully completing the required number of mPCP sessions specified in the manual; number of those dyads where there was a mutually agreed earlier termination, including reasons for early termination; early (non-agreed) dropouts from the intervention, including reasons for early dropout; (2) methods developed for measuring dyad treatment attendance and CAPT adherence to the manual, including the number of mPCP sessions offered, attended, and missed, and evidence of the appropriate use of the clinical approach advocated in the intervention manual; (3) preliminary assessment of the acceptability of the intervention, including negative outcomes, serious adverse events (SAEs), and related and unexpected SAEs (RUSAEs) (all RUSAEs occurring from the date of consent up to 4 (8, where applicable) months post randomisation will be recorded, reviewed by the chief investigator, and reported to the Research Ethics Committee (REC) and the sponsor within 15 days; (4) mapping of the range of standard care (CAMHS/external) pathways across arms; and (5) number of patients where the standard care pathway is known.

Follow-up data collection: (1) proportion of dyads with self-reported outcome data, proportion obtained at 4 months (and 8 months if applicable); (2) proportion of dyads with teacher-reported outcome data obtained at baseline and 4 months; (3) proportion of dyads with self-reported health economic data at 4 months; (4) proportion of dyads for whom it was possible to collect routine data, if this is pursued; (5) missing-item-level data on self-reported questionnaires; and (6) number of unblinding events of trial researchers over the data collection periods.

Statistical outcomes: (1) variability of self-reported outcome measures at baseline, 4 months; (2) clustering effect (ICC – intracluster correlation coefficient) relating to CAPTs in the intervention arm; (3) difference in self-reported outcomes available at 4 months per treatment arm; and (4) 95% confidence intervals (CI) for differences in outcomes between arms.

Health economic outcomes: (1) variability of the self-reported and proxy-reported EQ-5D-Y at baseline and 4 months; (2) completeness of and ability to obtain resource use data from dyads about both NHS and private costs; (3) exploratory cost-effectiveness analysis; and (4) suitability of a decision analysis model and appropriate time horizon to assess cost-effectiveness in the confirmatory RCT.

Process evaluation outcomes: (1) understanding of the recruitment process (with ideas for improving recruitment); (2) views on the intervention/trial; (3) reasons for declining to take part, dropping out of treatment, or withdrawing from the trial; (4) understanding of the contextual factors affecting involvement in the trial; (5) understanding of the barriers to, and enablers for, implementation; and (6) understanding of possible mechanisms of change.

Qualitative: establishment of (1) processes for successful recruitment of CAPTs; (2) procedure for training of CAPTs; (3) method for agreeing CAPT competence; and (4) procedure for ongoing supervision of CAPTs.

### Monitoring treatment attendance and adherence

Data will be collected on child and primary carer attendance at sessions. The steps taken to ensure consistency in the use of the mPCP intervention will also be recorded (attendance at training and supervision sessions for CAPTs, adherence of CAPTs to the manual). Child and primary carer mPCP intervention sessions will be tape-recorded where consent for this has been obtained from the primary carer, with the assent of the child. CAPT supervision sessions will also be tape-recorded, with consent. The chief investigator will review a selection of the mPCP tape-recorded intervention and supervision sessions to ensure, and allow reporting of, overall adherence of CAPTs to the manual. We will develop a coding and scoring system for assessing CAPT adherence to the manual, with a global rating of overall adherence.

### Sample size

The trial is designed to determine the feasibility of a confirmatory trial, not to assess proof of concept or evaluate effectiveness. It is generally recommended for feasibility studies that the analysis data set comprises a minimum of 30 participants per arm [65–67]. However, based on a review of the academic literature [68–78], estimates of the standard deviation (SD) of CBCL total score are already available, indicating that it is reasonable to assume a common SD across arms. We therefore require a minimum of 30 evaluable dyads across the arms to confirm the SD. Loss to follow-up has ranged from 8 to 38%, so we need up to 50 dyads randomised to guarantee 30 evaluable dyads. Treatment attendance has ranged from 40 to 95%. Assuming that we can reduce loss to follow-up to 10% at 4 months, randomising 60 dyads on a 1:1 basis will allow us to estimate the pooled SD with a 95% CI with expected width of 0.39 multiplied by SD, loss to follow-up with a 95% CI with expected width ± 7%, and treatment attendance in the intervention arm with a 95% CI with expected width of between ± 8 to 18%. It is expected that 60 dyads will provide sufficient opportunity to establish robust recruitment and retention strategies for a confirmatory trial.

### Statistical analysis

The trial statisticians will be responsible for statistical analysis. The analysis plan outlined in this section will be reviewed and a final, more detailed statistical analysis plan will be written before any analysis is undertaken. Any changes to the finalised analysis plan and reasons for change will be documented. There are no planned subgroup analyses. No formal analyses are planned until after the trial is closed to recruitment and all patients have been randomised. Final analysis will be carried out when all available outcome data have been received.

The recruitment strategy will be evaluated by summarising the screening, eligibility, consent, and randomisation stages, clearly separating the child from their primary carer, including numbers of dyads involved during each stage. Reasons for non-participation in the trial will be summarised, dyad retention during follow-up, including number of dyads completing/withdrawing from the trial and reasons for withdrawal, will be presented overall and by treatment arm.

Receipt of mPCP will be evaluated by summarising the proportion of dyads attending at least one session and those successfully completing the required amount of mPCP as specified in the manual (or fewer, as determined by the CAPT), both individually and as a dyad. The number of sessions and pattern of attendance will also be summarised. The number of early mutually agreed terminations and non-agreed dropouts from the intervention arm will be reported and the reasons for dropout summarised, where available. The method for measuring mPCP treatment attendance by the dyad will be agreed and summarised, i.e. number of sessions attended and length of sessions attended by child and primary carer, respectively, and a method for measuring manual adherence will be developed, including CAPT attendance at mPCP training, regular attendance and presentation at clinical supervision, development of an appropriate scoring system designed to assess manifestation by CAPTs of key components of the manual, review of a randomly selected subset of tape-recorded child and primary carer mPCP sessions and CAPT supervision sessions, employing this scoring system. The range of co-interventions and TaU (in CAMHS) in both arms will be summarised by treatment arm, as will referrals outside of CAMHS. The feasibility of obtaining information on care provided outside of the trial treatments will be assessed by the number of dyads for which this care is known, overall and by treatment arm.

The feasibility of obtaining the confirmatory trial’s primary and secondary outcome data will be assessed by summarising the proportion of dyads with available self-reported outcome data by method of obtainment, overall and by treatment arm. Levels of, and where possible predictors of, missing self-reported outcome data, both at the individual-item level and for entire outcome measures, will be reported overall and by treatment arm.

In order to inform the sample size/power calculation for the confirmatory trial, we will assess the variability (SD) of the CBCL total score in the intervention and control arms to confirm the SD to be used. The clinically relevant difference will be assessed by establishing the mean scores (and corresponding 95% CIs) of the CBCL total score in the control arm and by seeking clinical opinion. Point and interval estimates of the eligibility, recruitment, and treatment attendance rates in this feasibility trial, along with the number of participants successfully followed up, will also help to inform the sample size by allowing estimation of the likely loss to follow-up rates that will occur, and will allow assessment of the feasibility of recruitment for the confirmatory trial. An investigation of the clustering effect (ICC) relating to the CAPTs will be carried out to aid sample size calculation. The difference in outcomes between the control and intervention arms at each point will be reported, with 95% CIs constructed around these differences. No formal testing will be done on this data.

### Health economic analysis

The health economist will be responsible for health economic analysis. We will evaluate the completeness of data and rate of missing data on individual questions to inform the methodology and the perspective to be used within the confirmatory trial. Although the cost-effectiveness analysis at this stage will only be exploratory, the incremental cost-effectiveness of a second-line mPCP intervention compared to TaU for children aged 5–11 years with treatment-resistant CD and their primary carers will be examined. We will also calculate indicative costs for the interventions.

Utility weights will be obtained from the EQ-5D-Y data as reported by the child or by the primary carer as a proxy-respondent for the child and collected as part of the feasibility trial follow-up. We will also consider aggregating utility gains, accumulating child and primary carer utility obtained from the EQ-5D. An exploratory cost-effectiveness analysis will be carried out on complete cases only (i.e. completion at all time points) and with imputations (multiple imputations based on age, gender, and treatment), and a set of within-feasibility trial sensitivity analyses will be undertaken.

### Analysis for the process evaluation

A researcher with expertise in qualitative data analysis will be responsible for analysis of the process evaluation. Quantitative data from questionnaires will be analysed using descriptive statistics to describe participant characteristics, views on the intervention/trial, and reasons for declining to take part, dropping out of treatment, or withdrawing from the trial. Qualitative data will be analysed inductively using thematic analysis [79, 80] and coded independently by two researchers for emerging themes. The researchers will then compare codes and themes and resolve any discrepancies by consensus. The analysis will be further refined using constant comparative and contrastive approaches, and examination of negative cases. Data analysis will begin alongside data collection in order to refine and develop the interview topic guide(s) and sampling strategy, so that emerging themes can be explored in greater depth. This early data analysis will also help to identify when further data collection is unlikely to yield any significant new themes (i.e. ‘data saturation’ is reached), and so inform the number of primary carer/child interviews actually required. Results will be used to optimise the information provided to participants about the trial to improve recruitment and will inform the confirmatory trial design and intervention delivery, e.g. changes to recruitment procedures might be made in response to feedback from those declining to take part.

### Other analysis

Sessions from the intervention adherence monitoring might additionally be transcribed and subject to further exploratory analysis as part of MSc dissertation projects.

### Ethical considerations

Ethical approval was granted for the trial by Yorkshire and The Humber Bradford Leeds Research Ethics Committee (Reference: 16/YH/0055). The right of participants to refuse participation without giving reasons will be respected and participants will remain free to withdraw from the trial at any time without giving reasons and without prejudicing further treatment. Primary carers will make any such decision on behalf of the child. The study protocol was written in line with the Standard Protocol Items: Recommendations for Interventional Trials (SPIRIT) 2013 Statement [81], provided here as Additional file 1.

### Trial governance

The Trial Management Group (TMG), comprising the chief investigator, trial manager, co-investigators, and CTRU team, will meet bi-monthly and will be responsible for the clinical set-up, ongoing management and interpretation of results. The Trial Steering Committee (TSC), with an independent chair, will meet 6-monthly and provide overall supervision of the project, including trial progress, adherence to protocol, safety, and consideration of new information.

### Public and Patient Involvement (PPI)

Primary carers of children with CD and parents with mental health difficulties provided input into the design of the trial and both they and children with CD provided input into trial-specific patient literature. Throughout the trial, we will work with a PPI lay advisory group (LAG) who will represent the needs of primary carers and children with CD in the ongoing conduct of the trial. Members of the LAG will also advise on promoting the trial and disseminating results, and will be involved in the interpretation of the qualitative results.

## Discussion

A series of success criteria have been set. If all of these are met, the trial will be considered feasible with no revisions needed. If they are not met and revisions are not possible, the trial will be considered unfeasible. If revisions are needed but are possible, the suggested revisions will be described in detail in the feasibility trial outputs. The success criteria are as follows: (1) recruitment is feasible, indicated by randomising to target; (2) treatment is acceptable, indicated by qualitative feedback from primary carers (and children, when possible) and from CAPTs; (3) attendance at the mPCP intervention is acceptable, indicated by child and primary carer, respectively, attending more than 50% of the anticipated 12 sessions offered; (4) data collection procedures for demonstrating manual adherence are feasible, indicated by sessions recorded, random subsets analysed, and qualitative feedback from CAPTs; (5) data collection burden is acceptable to primary carers, indicated by qualitative feedback; (6) missing data is at an acceptable level, indicated by 75% of visits completed and minimal non-completion of questionnaires within visits; and (7) estimates needed for the sample size calculation obtainable.

We anticipate six main challenges in the delivery of this project. First, outside of major centres, local community CAMHS are often overstretched clinically, and relatively inexperienced in participating in RCTs. Ongoing reviews of service provision and clinical pathways have already led to one service, initially enthusiastic to participate, withdrawing from the trial before its commencement, as local service reorganisation meant a significant reduction in capacity to provide a service to children with CD. Delivering manualised protocols against this backdrop will be a challenge and provide valuable information about the feasibility of the larger confirmatory trial.

Secondly, recruitment to the mPCP arm will be limited by CAPT availability. Although CAPTs are a core profession in CAMHS in the Children’s National Service Framework [82, 83], they are a relatively scarce resource in the majority of CAMHS, and are likely to have only very limited slots available for provision of the intervention. Moreover, CAPTs work on a specific time scale and do not usually commence an intervention less than 3–5 weeks prior to an end-of-term or half-term break in order to avoid early interruption to the treatment. This means that recruitment will need to occur within very specific time frames. We will seek to maximise CAPT availability through careful negotiation with participating CAMHS and by drawing on CAMHS where there is more than one CAPT, when possible. To avoid selection bias, we plan to randomly allocate CAPTs to dyads allocated to the intervention arm; however, doing so under the constraints of CAPT capacity, variable numbers of CAPTs per service, allocation of both child and primary carer, and against the trial’s relative priorities, may introduce further challenges.

A third challenge is likely to be retention of participants in treatment and/or in the research. As previously stated, treatment dropout rates for this patient group are likely to be high and collection of follow-up data might, therefore, also be affected. We will seek to address treatment dropout through the use of specific engagement strategies in the mPCP intervention, and to minimise loss to follow-up by emphasising to participants that dropping out of treatment does not mean withdrawing from the research.

Fourthly, with regard to the trial intervention, the design and length of mPCP has inevitably been affected by financial limitations, and the majority of CAPTs are not experienced with the use of manualised interventions. In non-manualised PCP, CAPTs would usually expect to see children and their primary carers for a longer (and, therefore, more expensive) period. There is, therefore, a question as to whether a confirmatory RCT would be a fair trial of treatment. However, weekly work for primary carers is often now the exception rather than the rule (except when CAPTS are in training, when it is more common), and, therefore, the intensity of the intervention might mitigate the shortness of its duration.

Fifthly, identification of reliable, valid, non-interview-based measures of attachment that can be practically used in an RCT with limited finances is challenging [84]. Nevertheless, there are some early indications in the academic literature that parental reflective functioning might correlate with child and adult attachment.

Finally, a ‘sleeper effect’ is often claimed for PCP [85], and NICE has indicated that research is needed on longer-term outcomes [1]. We would, therefore, ideally like a minimum of 1-year follow-up in a confirmatory trial. Given the financial constraints of this feasibility study, however, we have opted for partial follow-up at 8 months post randomisation for the initial cohorts, with a view to assessing the feasibility of longer-term data collection.

A recent systematic review and meta-analysis evaluating the efficacy of non-pharmacological interventions for CD in children and adolescents has found that most previously conducted RCTs are of very poor to fair quality with small effect sizes [86]. It concludes that there is insufficient evidence to support any one psychological treatment (including parenting programmes) over another for this patient group, and that future studies need to provide detailed information about randomisation and blinding arrangements, to triangulate parent-reported measures, and to use full ITT analysis. There are currently no ongoing or planned RCTs evaluating mPCP for this patient group. The current trial is, therefore, an important step towards evaluating the effectiveness of a specific type of intervention (mPCP) not usually researched by means of RCTs, and in investigating a much-needed, second-line intervention for this hard-to-reach patient group.

### Trial status

Recruitment of participants is ongoing in two trial districts: one in Yorkshire, centred on the cities of Bradford, Sheffield, and Wakefield, and the other in the West Midlands, centred on Dudley and Walsall.

## Additional file

Additional file 1: SPIRIT Checklist. (DOC 120 kb)

## Abbreviations

ACP: Association of Child Psychotherapists
ADHD: Attention deficit hyperactivity disorder
ASD: Autistic spectrum disorder
AUDIT-C: Alcohol-Use Disorders Identification Test
BNF: *British National Formulary*
CAMHS: Child and Adolescent Mental Health Service
CAPT: Child and adolescent psychotherapist
CBCL: Child Behavior Checklist
CBCL-TRF: Child Behavior Checklist-Teacher Report Form
CD: Conduct disorders
CI: Confidence interval
CNA: Could not attend
CTRU: Clinical Trials Research Unit
CYP-IAPT: Children and Young People’s Improving Access to Psychological Therapies
DAST-10: Drug Abuse Screening Test
DNA: Did not attend
EQ-5D: EuroQol 5 Dimensions
EQ-5D-Y: EuroQol 5 Dimensions Youth
FSF: Flexibility and Sustainability Funding
GAF: Global Assessment of Functioning
GHQ-12: General Health Questionnaire
GP: General practitioner
HCAM: Hampstead Child Adaptation Measure
HRA: Health Research Authority
ICC: Intracluster correlation coefficient
IS-CA: Impairment Score for Children and Adolescents
ISRCTN: International Standard Randomised Controlled Trial Number
ITT: Intention-to-treat
LAC: Looked After Child
LAG: Lay advisory group
LD: Learning difficulties
mPCP: Manualised Psychoanalytic Child Psychotherapy
NHS: National Health Service
NICE: National Institute for Health and Care Excellence
NIHR: National Institute for Health Research
PCP: Psychoanalytic Child Psychotherapy
PPI: Patient and Public Involvement
PSI: Parenting Stress Index
PSSRU: Personal Social Services Research Unit
QALY: Quality-adjusted Life Year
RCT: Randomised controlled trial
REC: Research Ethics Committee
RfPB: Research for Patient Benefit
RUSAE: Related and unexpected serious adverse event
SAE: Serious adverse event
SD: Standard deviation
TaU: Treatment as Usual
TIGA-CUB: Trial on Improving Inter-Generational Attachment for Children Undergoing Behaviour problems
TMG: Trial Management Group
TSC: Trial Steering Committee.
